# Linezolid and Meropenem for *Nocardia otitidiscaviarum* Actinomycetoma, India

**DOI:** 10.3201/eid3109.250514

**Published:** 2025-09

**Authors:** Kabir Sardana, Savitha Sharath, Soumya Sachdeva, Shukla Das, Gargi Rai, Praveen Kumar Singh

**Affiliations:** Atal Bihari Vajpayee Institute of Medical Sciences and Research Institute and Dr Ram Manohar Lohia Hospital, New Delhi, India (K. Sardana, S. Sharath, S. Sachdeva); University College of Medical Sciences and Guru Teg Bahadur Hospital, New Delhi (S. Das, G. Rai, P.K. Singh)

**Keywords:** mycetoma, bacteria, antimicrobial resistance, actinomycetoma, linezolid, meropenem, Nocardia otitidiscaviarum, refractory, relapse, treatment, sensitivity, combination, India

## Abstract

Treatment of actinomycotic mycetoma with joint involvement is challenging. We present a patient in India with actinomycotic mycetoma who reached complete cure and remission after linezolid and meropenem treatment with a 2-year posttreatment follow-up. Clinicians should use novel drug regimens based on subspecies variations of *Nocardia* and regional drug susceptibility patterns to guide therapy.

Actinomycotic mycetoma presents as a triad of symptoms: painless swelling, discharging sinuses, and presence of grains. *Nocardia brasiliensis* is commonly implicated (*1*). Although varied drugs and regimens have been tried, regional variations in antimicrobial drug sensitivities and species should guide therapy (*2*). We detail the clinical course of a refractory case caused by *N. otitidiscaviarum* infection in a patient in which complete clinical and radiologic remission was achieved with a combination regimen of linezolid and meropenem co-administered with trimethoprim/sulfamethoxazole. We also examine the role of penems on the basis of existing data.

In 2019, we saw a 30-year-old man for painless swelling and multiple pus-discharging sinuses in his right knee for 2 years, which was preceded by trauma from a road traffic accident. Actinomycotic mycetoma was diagnosed and treated with trimethoprim/sulfamethoxazole and dapsone for 6 months, which led to remission for 1 year before recurrence. The patient declined amikacin injections and was treated with trimethoprim/sulfamethoxazole (160 mg/800 mg 2×/d) and faropenem (300 mg) for 6 months, which resulted in complete resolution. Recurrence occurred within 10 months of stopping therapy.

On examination, multiple nodules with overlying sinuses and scanty seropurulent discharge were apparent on the anterior aspect of right knee (Figure 1, panel A). Ultrasonography of the right knee showed a characteristic dot-in-circle sign (Appendix Figure 1) that was confirmed by magnetic resonance imaging. A deep incisional skin biopsy specimen from the nodule revealed epidermal hyperkeratosis, parakeratosis, neutrophil exudate, and irregular acanthosis with multiple small grains in superficial dermis rimmed by dense neutrophilic infiltrate, suggestive of Splendore-Hoeppli phenomenon. 

**Figure 1:**
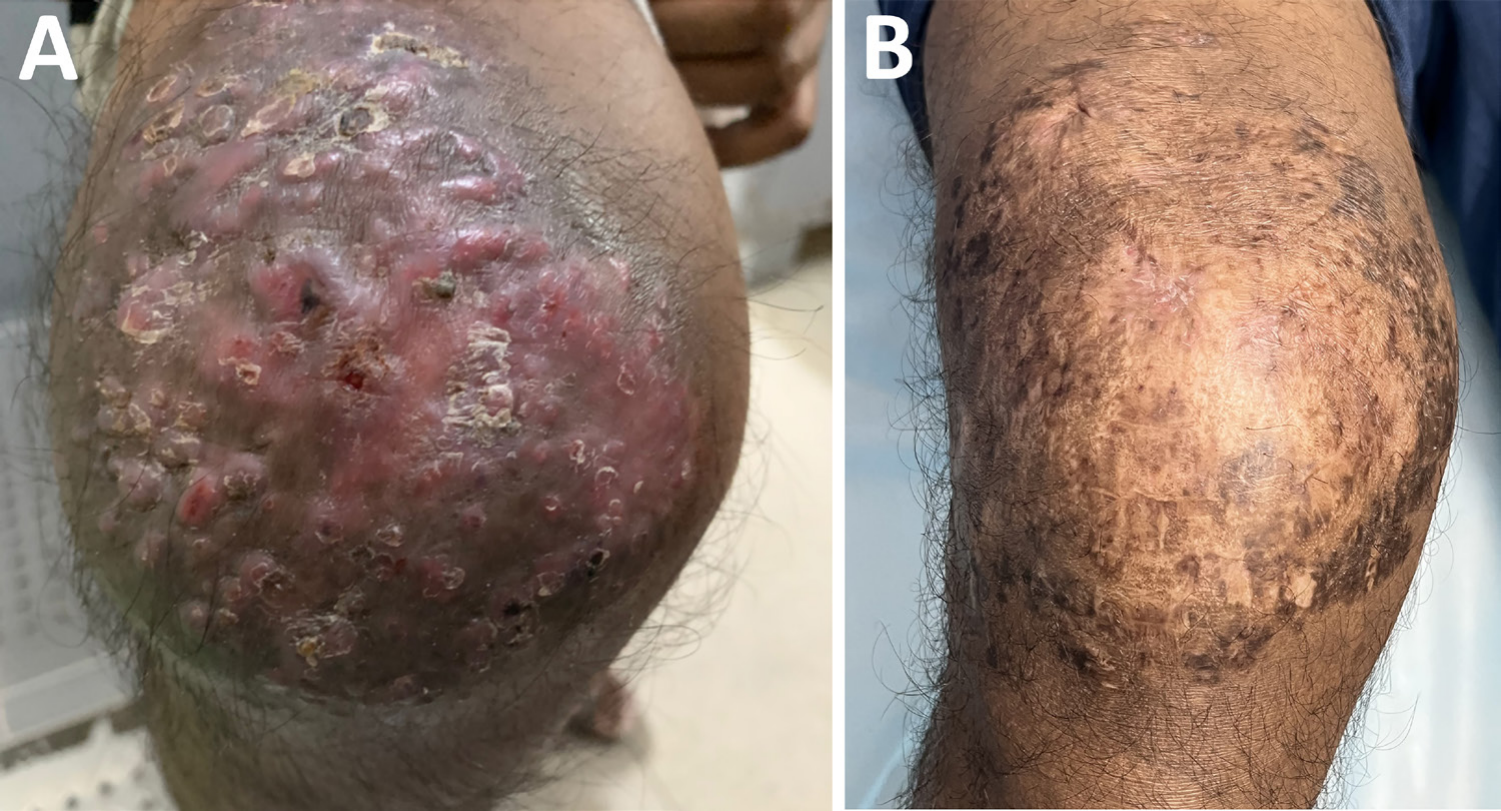
Anterior right knee of patient treated with linezolid and meropenem for *Nocardia otitidiscaviarum* actinomycetoma, India. A) Multiple nodules with overlying sinuses over right knee. B) Near-complete resolution with subsidence of sinuses and nodules leaving behind scarring after 2 months of treatment with a 21-day cycle of linezolid and meropenem combined with trimethoprim/sulfamethoxazole.

The biopsy specimen collected from the nodule on the right knee revealed the presence of gram-positive, thin, branching filaments, suggestive of actinomycetes. Modified Ziehl-Neelsen stain (using 1% sulphuric acid) showed acid-fast, thin, branching, beaded, filamentous bacilli. Blood agar showed growth of colonies 2–3 mm in size after 72 hours of aerobic incubation that appeared dry, convex, white, and adherent to the medium (Figure 2). On Sabouraud dextrose agar, the colonies were dry and yellowish-orange in color. Subculture and microscopy revealed branching gram-positive rod, and *Nocardia* was confirmed.

**Figure 2:**
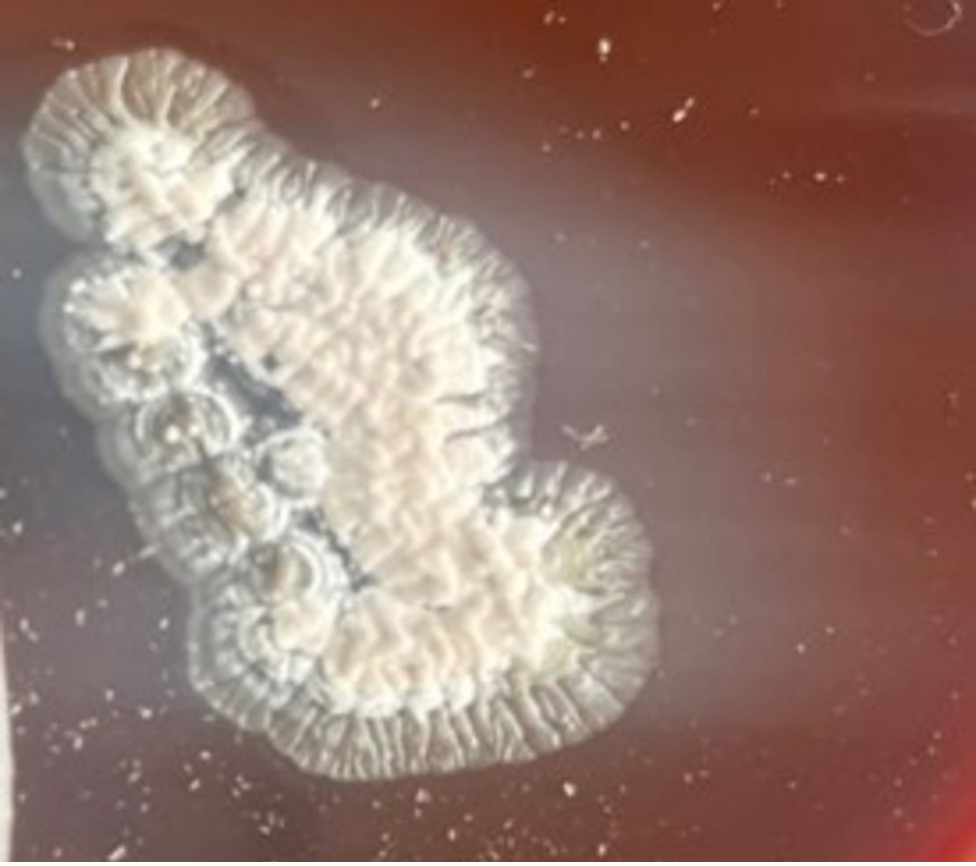
Tissue culture from patient treated with linezolid and meropenem for *Nocardia otitidiscaviarum* actinomycetoma, India. Blood agar culture shows growth of dry, convex, white colonies that are adherent to the medium. Original magnification ×200.

Sequencing using found on PCR displayed >99% similarity with *N. otitidiscaviarum* sequences deposited in GenBank (accession nos. NR_041874.1, KM678016.1, and OQ034626.1) by using BLAST (https://blast.ncbi.nlm.nih.gov) (Appendix). A sensitivity assay using a Sensititre Rapid Growing Mycobacteria RAPMYCOI Plate (Thermo Fisher Scientific, https://www.thermofisher.com) showed sensitivity to sulfamethoxazole, ciprofloxacin, moxifloxacin, cefoxitin, amikacin, doxycycline, linezolid, imipenem, tobramycin, and ceftriaxone.

A 21-day cycle of intravenous linezolid (600 mg 2×/d) and meropenem (500 mg 3×/d), along with trimethoprim/sulfamethoxazole (160 mg/800 mg 2×/d) led to a decrease in discharge within 3 weeks and substantial clinical improvement within 2 months of treatment (Figure 1, panel B). Trimethoprim/sulfamethoxazole was continued at the same dose for another 10 months and stopped. Residual disease was treated with another cycle of linezolid and meropenem after 10 months. The patient tolerated therapy well and was in remission for 24 months after stopping treatment.

Actinomycotic mycetoma is primarily caused by *Nocardia*, *Streptomyces*, and *Actinomadura* species, and the highest incidence is reported in India, Asia, Pakistan, and Yemen (*1*). Of the various *Nocardia* species, *N. otitidiscaviarum* is rarely reported, predominantly affecting immunocompromised hosts (*3*), and is an uncommon and unreported cause of mycetoma. A previous study with DNA sequencing of 441 *Nocardia* species reported *N. otitidiscaviarum* in 5.9% of samples (*4*). Although there are reports of nocardiosis caused by that species (*4*), actinomycotic mycetoma has not been reported. The existing drug regimens entail cyclical administration and long durations of therapy, and our aim was to explore the use of penems as monotherapy or in combination to treat actinomycotic mycetoma (*2*,*5**,**6*), but no previous study has used a combination with linezolid.

We tried to avoid the use of amikacin because of its side effects. One the basis of sensitivity patterns and previous data (*7*), we used a combination of linezolid and meropenem. Although the sensitivity analysis was tested for imipenem, in vitro studies have shown higher activity of meropenem compared with imipenem against *Nocardia* and reflect its clinical efficacy (*8*). Linezolid has also shown in vitro activity against *Actinomadurae* spp. and *Nocardia* spp. in refractory actinomycotic mycetoma (*9*).

The refractory nature and recurrences in this case could be a consequence of *N. otitidiscaviarum* infection, which is an uncommon cause of actinomycotic mycetoma. The rapid response and long-term remissions make the described regimen suitable and saves inpatient admission costs and repeated admissions that are needed for other regimens (*10*). Thus, it is useful to collate existent sensitivity data with regional antimicrobial sensitivity for a logical combination regimen, and more data on that combination can determine its widespread applicability in mycetoma caused by *Nocardia* spp. Clinicians should use advances in drug regimens according to subspecies variations of *Nocardia* and regional antibiotic drug susceptibility patterns to guide therapy.

Appendix Additional information for linezolid and meropenem for *Nocardia otitidiscaviarum* actinomycetoma.
