# Supplementary material for: Linezolid and Meropenem for Nocardia otitidiscaviarum Actinomycetoma, India
Source: Emerg Infect Dis. 2025 Sep;31(9):1863–5. doi: 10.3201/eid3109.250514 (PMC12407198; doi:10.3201/eid3109.250514)
Supplement: Appendix — Additional information for linezolid and meropenem for Nocardia otitidiscaviarum actinomycetoma. [file 25-0514-Techapp-s1.pdf]

Article DOI: https://doi.org/10.3201/eid3109.250514

EID cannot ensure accessibility for supplementary materials supplied by authors. Readers who have difficulty accessing supplementary content should contact the authors for assistance.

Linezolid and Meropenem for *Nocardia otitidiscaviarum* Actinomycetoma

Appendix

Sequencing Using Polymerase Chain Reaction

The collected tissue biopsy was further processed for DNA extraction using DNA extraction kit (HiYield Genomic DNA Kit, red blood cell, Taiwan). Subsequently, the extracted DNA was subjected to polymerase chain reaction (PCR) amplification using primers specific for the 16S rDNA of genus Nocardia, NG1: 5′-ACCGACCACAAGGGGG-3′ and NG2: 5′-GGTTGTAAACCTCTTTCGA-3′. PCR amplification was carried out in a Mastercycler personal (Eppendorf) with initial denaturation of DNA at 95°C for 5 min, followed by 35 cycles consisted of a denaturation at 95°C for 30 sec, annealing at 55°C for 30 sec, extension at 72°C for 45 sec, and a final extension at 72°C for 10 min. The PCR amplicons were resolved on 2% agarose gel with 0.5mM ethidium bromide and visualized on UV transilluminator. A band of ≈600 bp (bp) was obtained (Appendix Figure 2). The sequencing of positive PCR product was done commercially using Sanger technology. The sequence was compared with sequences deposited in GenBank using BLAST alignment software (http://www.ncbi.nlm.nih.gov/BLAST).

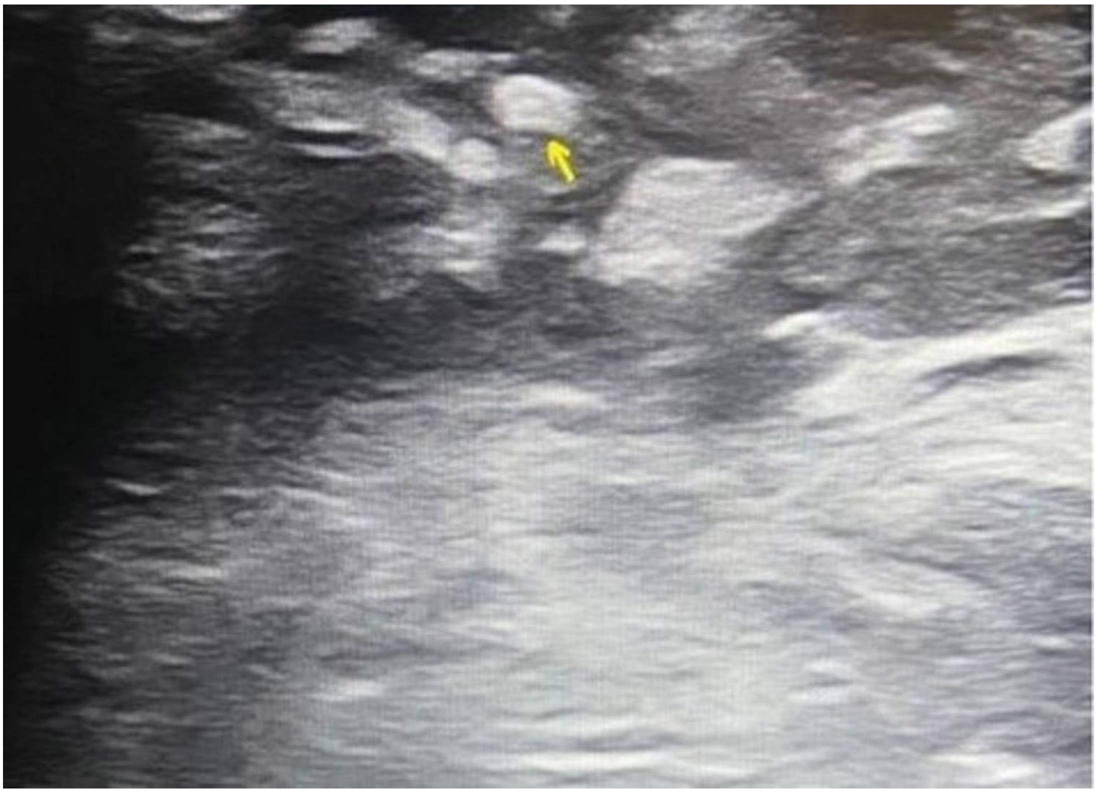

Appendix Figure 1. Ultrasound of right knee showing the dot in circle sign (yellow arrow).

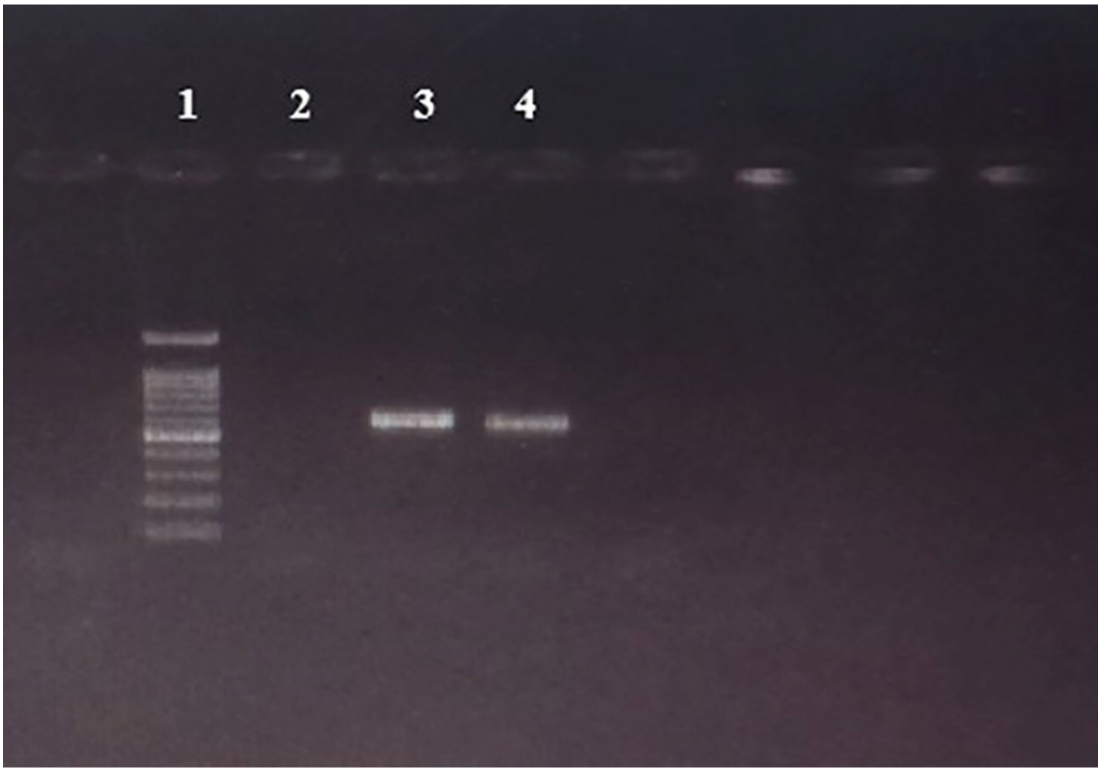

Appendix Figure 2. Gel picture showing amplicons of *Nocardia otitidiscaviarum* using NG1 and NG2 primers. Lane 1: 100-bp DNA ladder; Lane 2: Negative control; Lane 3: Positive Control; Lane 4: Positive for *Nocardia* at 600 bp.
